# Surfactin accelerates *Bacillus subtilis* pellicle biofilm development

**DOI:** 10.1016/j.bioflm.2024.100249

**Published:** 2024-12-30

**Authors:** Rune Overlund Stannius, Sarah Fusco, Michael S. Cowled, Ákos T. Kovács

**Affiliations:** a DTU Bioengineering, Technical University of Denmark, 2800, Kongens Lyngby, Denmark; b Institute of Biology Leiden, Leiden University, 2333, BE, Leiden, Netherlands

**Keywords:** *Bacillus subtilis*, Biofilm, Secondary metabolite, Surfactin, Development

## Abstract

Surfactin is a biosurfactant produced by many *B. subtilis* strains with a wide variety of functions from lowering surface tension to allowing motility of bacterial swarms, acting as a signaling molecule, and even exhibiting antimicrobial activities. However, the impact of surfactin during biofilm formation has been debated with variable findings between studies depending on the experimental conditions.

B. subtilis is known to form biofilms at the solid-air, the solid-medium, and the liquid-air interfaces, the latter of which is known as a pellicle biofilm. Pellicle formation is a complex process requiring coordinated movement to the liquid-air interface and subsequent cooperative production of biofilm matrix components to allow robust pellicle biofilm formation. This makes pellicle formation a promising model system for assaying factors in biofilm formation and regulation.

Here, we assayed the influence of surfactin and additional metabolites on the timing of pellicle biofilm formation. Using time-lapse imaging, we assayed pellicle formation timing in 12 *B. subtilis* isolates and found that one, MB9_B4, was significantly delayed in pellicle formation by approximately 10 h. MB9_B4 was previously noted to lack robust surfactin production. Indeed, deletion of surfactin synthesis in the other isolates delayed pellicle formation. Further, pellicle delay was rescued by addition of exogeneous surfactin. Testing reporters of biofilm-related gene expression revealed that induction of pellicle formation was caused by a combination of increased gene expression of one of the biofilm components and promotion of growth.

## 1 Introduction

Biofilm is one of the most common lifestyles of bacteria in natural settings [[1], [2], [3], [4]]. During biofilm formation, microorganisms produce a robust extracellular matrix consisting of polysaccharides, proteins, and eDNA, which together provide protection against antibiotics, predators, invasion or non-cooperative microorganisms, and other environmental factors [[5], [6], [7], [8]]. Formation of biofilm is a complex coordinated effort in which spatial gene expression patterns [9,10], division of labor [11], and cell differentiation [12,13] are common, and these make biofilm development a highly relevant target within the field of sociomicrobiology [14,15]. Additionally, biofilm and the dynamics of their formation are highly relevant in our society as an agent of persistent bacterial colonization in sickness and in health of humans, animals, and plants, as well as industrial production and infrastructure [16].

Growing in a biofilm is a tradeoff with accompanying detrimental effects on the microbes within it; most biofilms are static, limiting space and nutrients; potentially negatively affecting fitness if formed indiscriminately in an ever changing environment [17,18]. Therefore, biofilm development is tightly regulated and tuned including precise gene regulatory pathways being activated during establishment, maturation, and disassembly toward a search for a new niche to colonize [19]. Biofilms are generally studied using distinct laboratory models, including architecturally complex colony biofilms at the interface between a solid substrate and air, submerged biofilms at the substrate-liquid interface, floating or host-embedded aggregates, and pellicle biofilms at the liquid-air interface [20]. These biofilm types have specific requirements to establish successfully, and therefore it is also important that the regulation of biofilm related processes either can accommodate several types of modalities or that biofilm itself is robustly functional in several situations.

Pellicle biofilms are in particular dependent on precise coordination to form a robust biofilm requiring coordinated movement and production of matrix to succeed [11,12]. Unlike colony biofilms, which can arise from clonal growth in one spot, cells during pellicle biofilm development transition from a planktonic phase in the liquid medium into a sessile state in the floating biofilm through a well-defined time window which leaves the lower liquid medium almost devoid of cells [14,21,22]. This behavior is easily observable and provides a good tractable model system for studying the factors determining biofilm formation and the coordination of differentiation.

*Bacillus subtilis* is a Gram-positive, soil-dwelling bacterium with well-documented plant growth promoting abilities through direct inhibition of plant pathogens as well as through induced systemic resistance and promotion of plant growth [23]. Plant protection by *B. subtilis* is mediated by a range of bioactive molecules such as lipopeptides which have been shown as a direct effector of plant growth and resistance. One such molecule is surfactin, a surface active agent involved in surface colonization by *B. subtilis* [[24], [25], [26], [27], [28], [29]] and abundantly produced during colonization of plant roots where it also induces systemic resistance [[30], [31], [32], [33], [34]]. Importantly, surfactin promotes *B. subtilis* to reach and colonize the rhizosphere when inoculated to the soil [35] and domesticated *B. subtilis* strains that lack surfactin production demonstrate reduced root colonization in the soil compared with undomesticated strains, unlike during direct inoculation of the strain on the seedlings in agar medium [36]. *B. subtilis* pellicle biofilm formation largely depends on two essential matrix components [37,38], exopolysaccharides (EPS) encoded by the *epsABCDEFGHIJKLMNO* operon (*epsA-O*) [39] and protein fibers of TasA, encoded by the *tapA-sipW-tasA* (*tapA*) operon [40].

While the importance of surfactin is clear in collective swarming motility and for its effects on plants, its impact on biofilm formation is controversial. A previous study found no significant difference in biofilm mass on tomato roots between wild-type strains and their surfactin biosynthetic gene cluster (BGC) deletion mutants in 5 *B*. *subtilis* root-derived isolates [41], others noted lack of biofilm formation and tomato colonization by the surfactin deletion mutant of *B. subtilis* 6051 [30]. Within the *B. subtilis* group species, *B. velezensis* (FZB42) and *B. amyloliquefaciens* (UMAF6614) were reported to have severe biofilm defects when surfactin biosynthesis operon was disrupted [42,43]. Indeed, disruption of surfactin biosynthesis was reported to have a species-dependent influence on pellicle formation in the *B. subtilis* group [44].

Previous reports demonstrated the direct influence of surfactin on induction of biofilm-related gene expression in the model strain *B. subtilis* NCIB3610 (hereafter 3610) when cells are grown in exponential growth phase; thus, practically non-biofilm inducing conditions [[45], [46], [47]]. Subsequent studies interpreted these results that surfactin is crucial for biofilm development of *B. subtilis*. Therefore, the essentiality of surfactin for biofilm formation has been previously revisited, which demonstrated that deletion of the surfactin biosynthetic gene *srfAA* in 3610 and six other recent isolates has no observable influence on pellicle formation, monitored after 20 h, and root colonization in biofilm inducing MSgg and MSNg media using hydroponic conditions [48]. Additionally, re-sequencing of the genome of the originally created and widely tested *srfAA* strain that displays reduced pellicle biofilm formation revealed additional point mutations, which likely explain disrupted biofilm development, especially, as re-introduction of the same *srfAA* mutation again into 3610 had no apparent influence on biofilm development after 20 h [48].

In this study, we dissect the influence of surfactin on biofilm development. Employing a time-lapse approach, we observed a significant delay of pellicle formation for the soil isolate MB9_B4, a recent natural *B. subtilis* isolate that lacks robust surfactin production. Furthermore, a similar delay was observed in other strains when surfactin production was removed, which was rescuable by addition of exogeneous surfactin.

Our data demonstrates that surfactin promotes an early development of pellicles in *B. subtilis*, validating the influence of surfactin on biofilm development, while not being essential for its pellicle formation.

## 2 Materials and methods

### 2.1 Strains, chemicals, and genetic modification

Strains used in this study can be found in Table 1 and have earlier been genomically and chemically characterized in [49]. Strains were routinely cultured in lysogeny broth (LB; Lennox, Carl Roth, 10 g/L tryptone, 5 g/L yeast extract and 5 g/L NaCl) and LBgm (LB supplemented with 1 % v/v glycerol and 0.1 mmol/L MnCl_2_, based on [50]). Antibiotics were used at the following final concentrations: Spectinomycin (spec) 100 μg/mL, kanamycin (kan) 5 μg/mL, tetracycline (tet) 10 μg/mL, chloramphenicol (chl) 10 μg/mL.

**Table 1:** Strains used in this study.

*B. subtilis strains*	Characteristics	Reference
DK1042	NCIB3610 *comI*^Q12L^, natural competent variant of NCIB3610	[55]
NRS2243	3610 *sacI*::P_*epsA*_-*gfp* (kan^R^)	[56]
NRS2394	3610 *sacI*::P_*tapA*_*-gfp* (kan^R^)	
DK1042 P_*srfAA*_-*gfp*	DK1042, *amyE*::P_*srfAA*_-*gfp* (chl^R^)	This study
TB501	DK1042, *amyE*::P_hyperspank_-*mKate2*, (spec^R^)	[11]
MB9_B4 mKate2	MB9_B4 transformed with gDNA from TB501 *amyE*::P_hyperspank_-*mKate2*, (spec^R^)	This study
MB9_B4 *P-epsA*	MB9_B4 mKate2 transformed with gDNA from NRS2243 *sacI*::P_*epsA*_-*gfp* (kan^R^)	This study
MB9_B4 *P-tapA*	MB9_B4 mKate2 transformed with gDNA from NRS2394 *sacI*::P_*tapA*_-*gfp* (kan^R^)	This study
MB9_B4 *P-srfAA*	MB9_B4 mKate2 transformed with gDNA from TB686.1 *amyE*::P_*srfAA*_-*gfp* (chl^R^)	This study
MB8_B1	natural isolate	[48]
MB9_B1	natural isolate	
P8_B1	natural isolate	
P9_B1	natural isolate	
P5_B1	natural isolate	
75	natural isolate with *amyE*::P_hyperspank_-*gfp* (chl^R^)	
MB8_B7	natural isolate	[49]
MB8_B10	natural isolate	
MB9_B4	natural isolate	
MB9_B6	natural isolate	
P8_B3	natural isolate	
73	natural isolate with *amyE*::P_hyperspank_-*gfp* (chl^R^)	
DTUB68	MB8_B1, *srfAC*::*Tn10* (spec^R^)	[48]
DTUB71	MB9_B1, *srfAC*::*Tn10* (spec^R^)	
DTUB80	P8_B1, *srfAC*::*Tn10* (spec^R^)	
DTUB82	P9_B1, *srfAC*::*Tn10* (spec^R^)	
DTUB79	P5_B1, *srfAC*::*Tn10* (spec^R^)	
DTUB89	75, *amyE*::P_hyperspank_-*gfp* (chl^R^) *srfAC*::*Tn10* (spec^R^)	
DTUB69	MB8_B7, *srfAC*::*Tn10* (spec^R^)	[49]
DTUB70	MB8_10, *srfAC*::*Tn10* (spec^R^)	
DTUB72	MB9_B4, *srfAC*::*Tn10* (spec^R^)	
DTUB73	MB9_B6, *srfAC*::*Tn10* (spec^R^)	
DTUB81	P8_B3, *srfAC*::*Tn10* (spec^R^)	
DTUB88	73, *amyE*::P_hyperspank_-*gfp* (chl^R^) *srfAC*::*Tn10* (spec^R^)	

*gfp: gfpmut2*, kan^R^: kanamycin resistance, chl^R^: chloramphenicol resistance, spec^R^: spectinomycin resistance.

All strains were naturally competent and genetically engineered using a modified version of the transformation protocol described in [51] to construct the biofilm-matrix and surfactin promoter fusion strains (P_*tapA*_-*gfp,* P_*eps*_-*gfp,* P_*srfAA*_-*gfp*) with constitutive red fluorescence (*amyE*::P_hyperspank_-*mKate2*). Briefly, 1 mL of overnight culture was spun down and resuspended in 100 μL sterile mili-Q water of which 10 μL were inoculated into 2 mL competence medium (80 mmol/L K_2_HPO_4_, 38.2 mmol/L KH_2_PO_4_, 20 g/L glucose, 3 mmol/L Na_3_-citrate, 45 μmol/L ferric NH_4_-citrate, 1 g/L casein hydrolysate, 2 g/L K-glutamate, 0.335 μmol/L MgSO_4_·7H_2_O), and incubated at 37 °C for 3.5 h. Donor DNA was extracted from 1 mL of overnight culture grown in LB using the Bacterial and Yeast Genomic DNA Purification Kit from EURx with a typical purified DNA concentration ranging from 50 to 150 ng/μL. 2 μl donor DNA was added to a new tube and washed down using 400 μL of competent cells and incubated for further 2 h before plating 100 μL on selective LB agar medium, which were subsequently incubated at 37 °C overnight to select for successful transformants.

The P_*srfAA*_-*gfp* promoter fusion for monitoring the expression of the biosynthetic gene cluster of surfactin was constructed in DK1042 (NCIB 3610 *comI*^Q12L^). Briefly, the P*rrnB* was replaced with the *srfAA* promoter in the pGFP-rrnB vector [52] using prolonged overlap extension PCR. The *srfAA* promoter region was PCR-amplified with the primers srfAA_forward (5' AGCTGTCAAACATGAGAATTGAAA GAATCGTTGTAAGACGC 3') and srfAA_reverse (5' AGTTCTTCTCCTTTGCTAGCTTATTTCCATATTGTC ATACCTCC 3'). pGFP-rrnB was linearized via PCR with pGFP_forward (5' GTATGACAATATGGAAA TAAGCTAGCAAAGGAGAAGAACT 3') and pGFP_reverse (5' CGTCTTACAACGATTCTTTCAATTCTCAT GTTTGACAGCTT 3') using Q5 DNA polymerase. The PCR products were directly transformed into *Escherichia coli* cells, and correct insert was verified by sequencing. The construct was introduced into *B. subtilis* DK1042 *amyE* locus using natural competence selecting for chloramphenicol resistance and verified by sequencing.

### 2.2 Culture conditions and pellicle formation timing

For pellicle timing assays, overnight incubated cultures in LB medium were adjusted to an optical density of 0.1 at 600 nm (OD_600_) using LB medium and 10 μl were inoculated into 1 mL of LBgm in 24-well microtiter plates and were incubated at 30 °C without shaking and followed using the ReShape imaging system (ReShape biotech, Denmark) which allowed time-lapse imaging of each microtiter well every 30 min over a period of 48 h. 10 μl of either surfactin dissolved in methanol (final concentration 20 μmol/L in the medium) or pure methanol was added to test the induction by surfactin. The study did not include any technical replicates as the differences between these often were lost due to the time between imaging. Biological replicates were the result of independent runs performed on different days. Six biological replicates were carried out for assaying pellicle timing for wild-type and Δ*srfAC* while three were performed for assaying induction by surfactin.

To set a definition for when a pellicle is formed, we manually annotated pellicle timing as in [53], defining pellicle formation timing as the, often sudden, transition from an empty mid-well to an opaque film covering the whole surface of the well. Pellicle formation varies further with some strains forming a visible ring in the periphery of the well before collapsing inwards, and pellicles generally diverge visually after the initial film has formed, thus our reasoning to time pellicle formation by the emergence of the first film as the most consistent visual indicator. We then cross-referenced our annotations with image analysis data from the ReShape interface, observing that well opacity was a possible marker for pellicle formation due to the low cell density in static cultures before assembly at the liquid-air interface. The formation of the first layer coincided with the average RGB red value reaching 100 (Fig. S1), which we used in addition to manual confirmation for determining pellicle formation timing in the rest of the study.

### 2.3 Promoter fusions

To assay potential induction by surfactin, overnight cultures were adjusted to 0.01 at OD_600_ and 10 μL was inoculated into 188 μL media in a 96-well microtiter plate, and 2 μL methanol-dissolved surfactin (final concentration of 20 μmol/L in the medium) was added with pure methanol as control. Each 96-well plate included three technical replicates for each promoter fusion and treatment combination, and three biological replicates were performed on different days. Growth and promoter fusion expression was followed by microplate reader (BioTek Synergy HTX Multi-mode Microplate Reader). Plates were incubated at 30 °C with continuous shaking and the following were measured every 10 min for 48h: OD_600_, green fluorescence for promoter-GFP fusion, red fluorescence for constitutively expressed mKate2 (Optics position: Bottom, GFP: ex: 485/20 em: 528/20, Gain: 60, mKate2: Ex: 590/20, Em: 635/20, Gain 60).

## 3 Results

### 3.1 Soil isolates feature remarkably similar windows of pellicle formation timing and show variable dependence on surfactin

While previous testing of *B. subtilis* isolates revealed comparable biofilm development after 2 days of incubation [48], time lapse imaging showed that the soil isolate MB9_B4 displayed a delayed pellicle formation by several hours compared with the closely related co-isolate MB9_B1. Despite delayed pellicle formation, the mature pellicle of MB9_B4 did not show any signs of decreased robustness, therefore allowing a comparative study of the factors influencing the timing of pellicle biofilm formation. Previous characterization of the isolates demonstrated differential ability in lipopeptide production, with MB9_B4 lacking robust surfactin production compared with the other isolates [49].

**Fig. 1:**
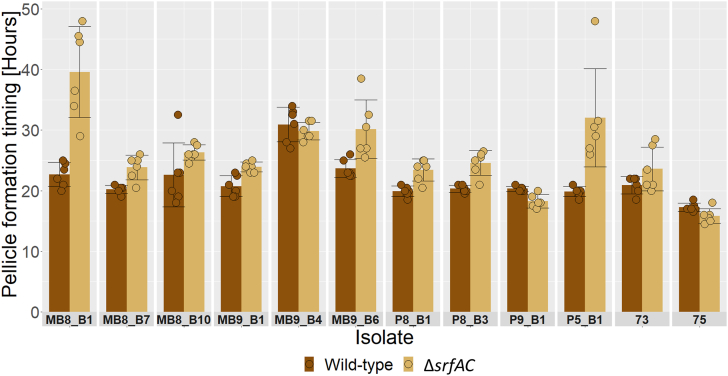
Average pellicle formation timing in LBgm at 30 °C for wild-type (dark brown) and Δ*srfAC* (beige) strains in rich biofilm inducing media with facets for each *B. subtilis* isolate separately. Average of 6 biological replicates, error bars represent standard deviation. (For interpretation of the references to colour in this figure legend, the reader is referred to the Web version of this article.)

**Fig. 2:**
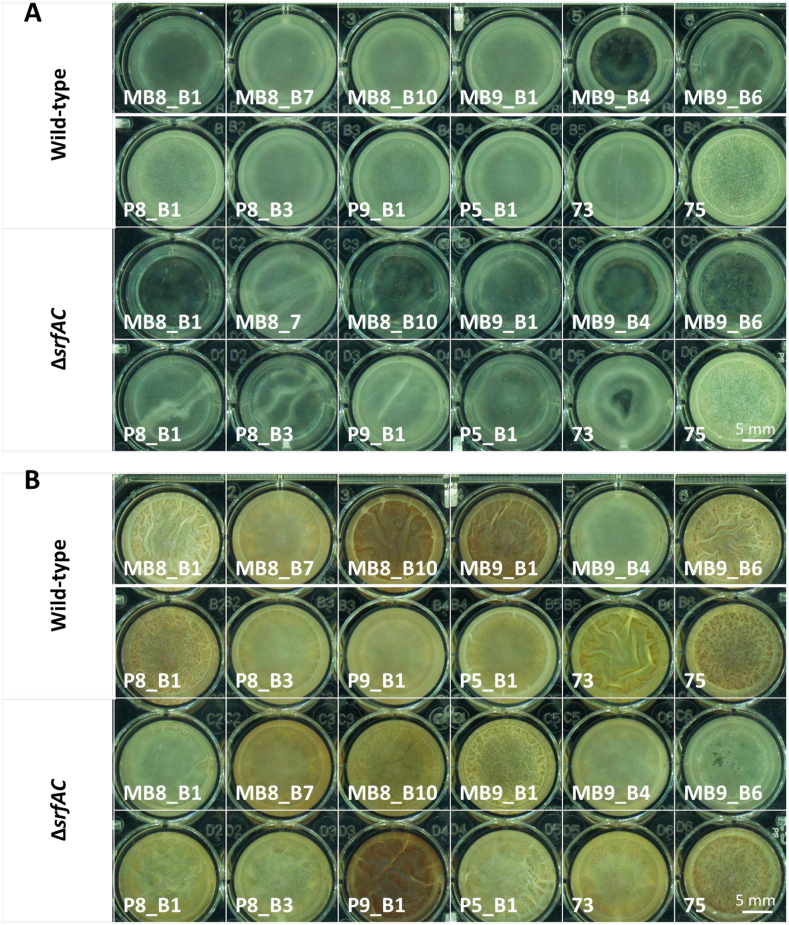
Pellicle biofilms cultured in 24-well plate at 30 °C, well diameter = 16.6 mm (5 mm scalebar shown in lower right corner of each 24-well plate). (A) Representative image from one of the six biological replicates showing pellicle cultures at 20 h showing early pellicles for most wild-type strains while the majority of Δ*srfAC* strains have not yet formed a pellicle. (B) Representative image of mature pellicle morphology at 48 h illustrating robust pellicle formation by all tested strains regardless of surfactin production.

Time-lapse imaging of various *B. subtilis* isolates and their derivative Δ*srfAC* mutants revealed that most of our strains formed pellicles within 20–25 h whereas MB9_B4 was delayed until 30.92 ± 2.84 h. Additionally, we found pellicle formation to be significantly delayed in 9 of the 12 Δ*srfAC* mutants compared to their respective wild-type ancestor (Fig. 1 and Table 2), indicating a role of surfactin in timing of pellicle formation in some strains. MB8_B10, MB9_B4, and 73 did not exhibit a significant difference between wild-type and Δ*srfAC* mutant, which for MB9_B4 might be explained by the isolate already lacking surfactin production, however, this is not the case for MB8_B10 or 73; thus, there may be other yet unknown factors that influence the timing of in pellicle formation (Fig. 2).

**Table 2:** Timings of pellicle formation, average of 6 biological replicates with ± denoting the standard deviation and Bonferroni adjusted P-values of pairwise t-tests between wild-type and Δ*srfAC* mutant for each strain, ∗ = below significance cut-off 0.05, NS = not significant.

Isolate	Pellicle timing, wild type	Pellicle timing, Δ*srfAC*	Difference	P-value
MB8_B1	22.67 ± 1.99	39.58 ± 7.52	16.92 ± 7.02	3.34E-04 ∗
MB8_B7	20.25 ± 0.69	23.83 ± 2.02	3.58 ± 1.93	2.08E-03 ∗
MB8_B10	22.58 ± 5.28	26.33 ± 1.25	3.75 ± 4.58	1.21E-01 **NS**
MB9_B1	20.75 ± 1.72	23.92 ± 0.80	3.17 ± 1.97	2.22E-03 ∗
MB9_B4	30.92 ± 2.84	29.83 ± 1.44	−1.08 ± 2.46	4.23E-01 **NS**
MB9_B6	23.67 ± 1.47	30.17 ± 4.83	6.50 ± 4.31	1.03E-02 ∗
P8_B1	19.92 ± 0.86	23.42 ± 1.80	3.50 ± 1.79	1.57E-03 ∗
P8_B3	20.33 ± 0.61	24.58 ± 2.04	4.25 ± 2.07	6.20E-04 ∗
P9_B1	20.33 ± 0.41	18.25 ± 1.08	−2.08 ± 1.28	1.32E-03 ∗
P5_B1	19.83 ± 0.82	32.00 ± 8.11	12.17 ± 8.18	4.40E-03 ∗
73	20.92 ± 1.43	23.58 ± 3.63	2.67 ± 5.02	1.25E-01 **NS**
75	17.25 ± 0.69	15.83 ± 1.21	−1.42 ± 0.92	3.20E-02 ∗

### 3.2 Exogeneous surfactin can advance and rescue pellicle formation timing

Our findings suggested that surfactin production is involved in pellicle formation timing. MB9_B4 is lacking robust surfactin production [49] and surfactin has been previously reported to induce expression of genes related to biofilm matrix production in various conditions [45,48]; thus, we next wanted to test if complementation was possible by supplementation of pure surfactin.

**Fig. 3:**
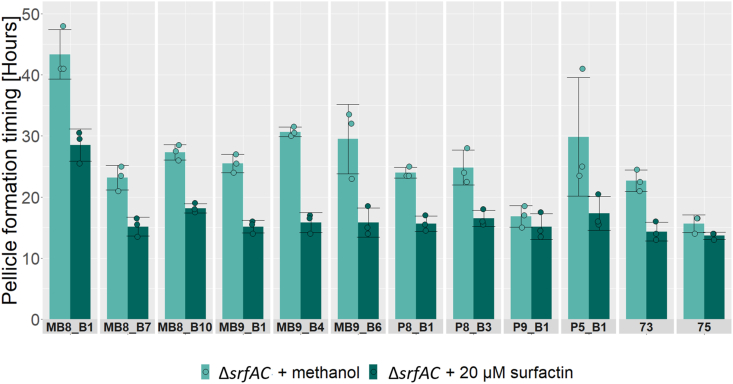
Induction of pellicle formation timing by addition of surfactin. Barplot showing average pellicle formation timing in LBgm at 30 °C for Δ*srfAC* treated with methanol (light teal) or 20 μM final concentration of surfactin (dark teal) with facets for each *B. subtilis* isolate separately. Average of 3 biological replicates treated with methanol or surfactin, error bars represent standard deviation.

Our results show that addition of 20 μM final concentration of surfactin was sufficient to ameliorate pellicle formation delay in MB9_B4 and furthermore significantly advanced pellicle formation timing in all but three of the tested Δ*srfAC* strains compared to that of the wild-type strain (Fig. 3 and Table 3).

**Table 3:** Timings of pellicle formation when treated with methanol (control) or 20 μM surfactin, average of 3 biological replicates with ± denoting the standard deviation and Bonferroni adjusted P-values from pairwise t-tests between pellicle timing of Δ*srfAC* control (pure methanol) and with added 20 μM final concentration of surfactin, ∗ = below significance cut-off 0.05, NS = not significant.

Strain (Δ*srfAC*)	Pellicle timing, Control	Pellicle timing, 20 μM surfactin	Difference	P-value
MB8_B1	43.33 ± 4.04	28.50 ± 2.65	14.83 ± 3.06	6.01E-03 ∗
MB8_B7	23.17 ± 2.02	15.17 ± 1.53	8. 00 ± 1.32	5.43E-03 ∗
MB8_B10	27.33 ± 1.26	18.17 ± 0.76	9.17 ± 1.04	4.19E-04 ∗
MB9_B1	25.50 ± 1.50	15.17 ± 1.04	10.33 ± 1.04	6.07E-04 ∗
MB9_B4	30.67 ± 0.76	15.83 ± 1.61	14.83 ± 1.26	1.34E-04 ∗
MB9_B6	29.50 ± 5.68	15.83 ± 2.36	13.67 ± 8.04	1.83E-02 ∗
P8_B1	24.00 ± 0.87	15.67 ± 1.26	8.33 ± 0.58	7.00E-04 ∗
P8_B3	24.83 ± 2.84	16.50 ± 1.32	8.33 ± 1.76	1.00E-02 ∗
P9_B1	16.83 ± 1.76	15.17 ± 2.08	1.67 ± 0.76	3.49E-01 **NS**
P5_B1	29.83 ± 9.70	17.33 ± 2.75	12.50 ± 6.95	9.83E-02 **NS**
73	22.67 ± 1.76	14.33 ± 1.53	8.33 ± 3.25	3.44E-03 ∗
75	15.67 ± 1.44	13.67 ± 0.58	2.00 ± 0.87	8.98E-02 **NS**

**Fig. 4:**
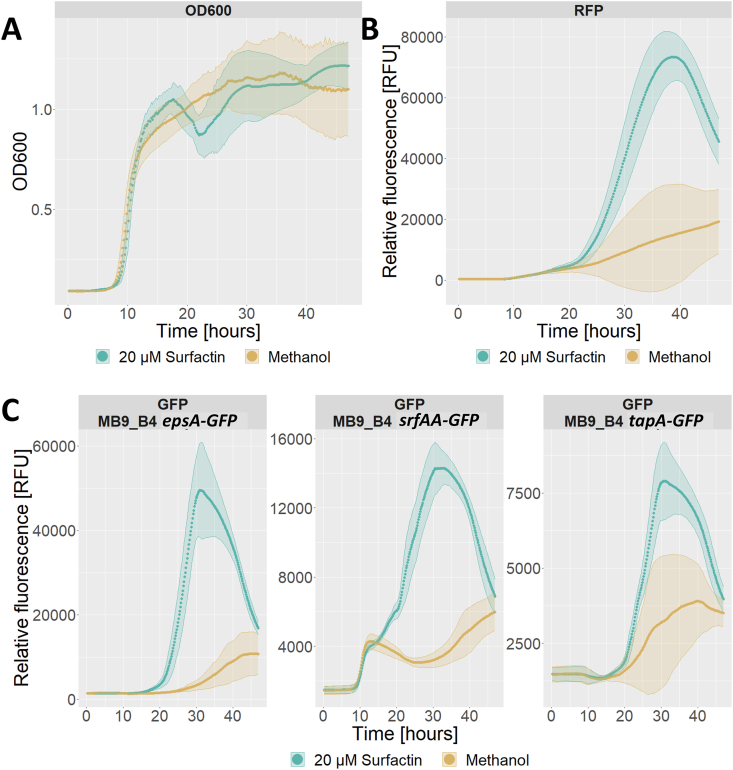
Effect of addition of surfactin to a final concentration of 20 μM (teal) or pure methanol as a control (beige) on growth and expression of fluorescence under constitutive and promoter-fusion coupled control in MB9_B4 over the course of 48 h in LBgm at 30 °C. A) Optical density at 600 nm. B) Red fluorescence from RFP under control of a constitutive promoter. C) Green fluorescence from GFP under control of promoter fusions for *epsA* (left), *srfAA* (middle), or *tapA* (right). Data from all three strains pooled for A) and B), while C) is separated for each promoter fusion (split figures can be found in Fig. S2). Data averaged from 3 biological replicates, each with 3 technical replicates with ribbons showing standard deviation. (For interpretation of the references to colour in this figure legend, the reader is referred to the Web version of this article.)

To understand the mechanism by which surfactin can hasten pellicle formation, we next assayed the effect of surfactin on the expression of biofilm matrix-related genes, *epsA* and *tapA* as well as on *srfA* itself using promoter fusions expressing GFP with an accompanying RFP tag under control of a constitutive promoter. Here, we observed little effect of surfactin on growth when judging by optical density at 600 nm (Fig. 4A), however, the constitutively active RFP tag seemed to exhibit increased fluorescence with addition of surfactin compared to the control (Fig. 4B), which could indicate increased growth. We do have to note that the RFP signal did not follow OD600 in early growth phases and might be an unreliable proxy for cell density, likewise, OD600 does not correlate well with cell density at higher densities which is where increased red fluorescence was observed. Additionally, an increase in green fluorescence from the reporter fusion which seemed independent from cell growth was observed for the *epsA* reporter fusion specifically with the addition of surfactin (Fig. 4C).

**Fig. 5:**
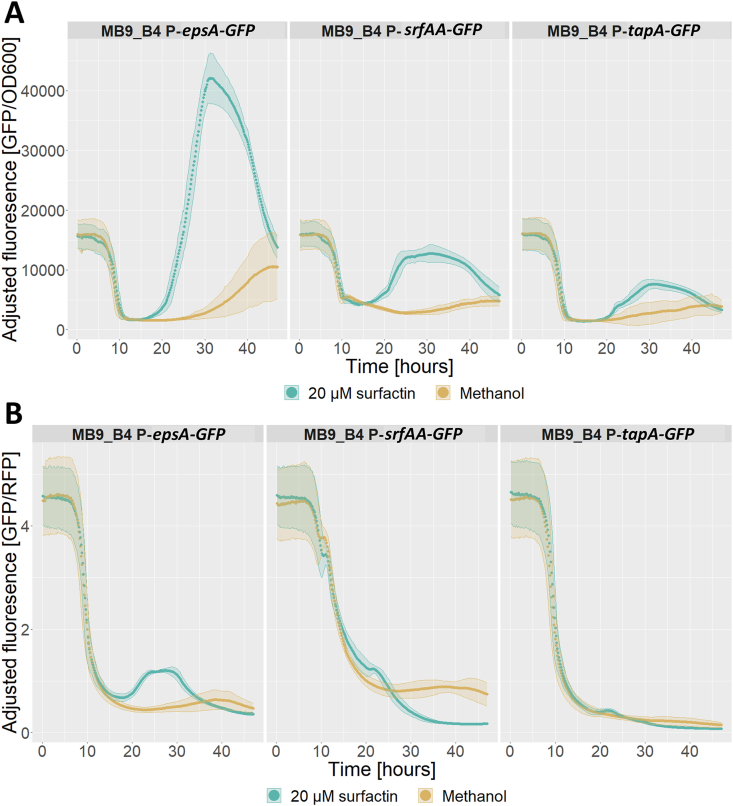
Adjusted reporter fusion expression of biofilm matrix-related genes *epsA* (left)*, tapA* (right), and surfactin biosynthesis connected *srfAA* gene (middle) in LBgm at 30 °C over the course of 48 h with either pure methanol (beige) or treatment with surfactin to a final concentration of 20 μM (teal) A) GFP fluorescence signal adjusted by optical density at 600 nm. B) GFP fluorescence signal adjusted by red fluorescence. Data is the average of 3 biological replicates, each with 3 technical replicates with ribbons showing standard deviation. (For interpretation of the references to colour in this figure legend, the reader is referred to the Web version of this article.)

To uncouple growth from gene expression, we adjusted GFP signal to account for live cell density by dividing the GFP signal value with either OD600 or the RFP signal. OD600 adjusted GFP signal showed an increase in expression of all three promoter fusions with the highest expression observed for *epsA* followed by *srfAA* and finally *tapA*, for all three promoter fusions, the increase in expression peaked around 30 h and decreased towards the end of the assay (Fig. 5A). The RFP adjusted GFP expression on the other hand, only displayed a short window of increased expression for *epsA* from 20 to 30 h and a reduction below the level of the control for *srfAA* from 25 h and on (Fig. 5B). Overall, these results indicate that surfactin might be affecting expression of some biofilm components, primarily *epsA* in the later stages of growth in biofilm inducing media.

## 4 Discussion

Revisiting the role of surfactin in induction of biofilm development, we sought to address the wide time-range at which pellicle formation is recorded. While some studies observed the presence of biofilms already after 8 h, other studies recorded pellicle formation after 48 h [44,45]; therefore, we employed time-lapse imaging to closely assay differences in biofilm development and induction of pellicle formation without a priori defining a time point when a pellicle should have been formed.

Due to our temporal resolution, we were able to precisely follow differences in pellicle formation of 12 *B. subtilis* soil isolates and their derivative Δ*srfAC* mutants, lacking surfactin production. We observed that all tested isolates were able to produce robust pellicles; however, one isolate, MB9_B4, which lacks robust surfactin production, displayed delay in pellicle formation production in rich biofilm inducing media. Furthermore, 9 out of 12 isolates were similarly delayed when their surfactin production capabilities were disrupted but were rescued by addition of exogeneous surfactin which advanced pellicle formation time sometimes below that of the wild-type for most strains. Promoter fusions reporting gene expression of genes involved in the synthesis of biofilm matrix components and surfactin biosynthesis further revealed that that surfactin might achieve pellicle timing advancement through induction of *eps* operon expression in addition to increased growth in late-stage cultures.

The observed induction patterns were different from those observed in earlier studies, in which different media were employed compared with our study that was based on LBgm medium. In [45], expression of *tapA* was only assayed in otherwise non-biofilm inducing LB medium, therein showing that surfactin (and nystatin) was able to increase *tapA* expression whereas our data only indicated low or no induction of *tapA* in the conditions used, i.e. LBgm medium. Meanwhile, in [48], the effect of a *srfAA* deletion on both *eps* and *tapA* expression were assessed in biofilm inducing media of MSgg and MSNc + pectin. Therein, promoter fusions indicated a correlation between *eps* expression and surfactin level in MSgg medium, unlike in MSNc + pectin medium, whereas *tapA* expression was reduced by surfactin in both MSgg and MSNc + pectin media. Finally, expression of biofilm related genes were recorded at the much earlier time points of 8 and 20 h in both previous studies, whereas we observed slightly increased induction after 15 h and majorly at 20 h and later.

Effects on growth and specific induction of gene expression are difficult to uncouple in the plate reader setup used here, thus, future experiments should include flow cytometry or microscopy to assess the effect on gene expression at single cells level. Additionally, it will be important to determine whether the influence of surfactin is uniform across the whole population or only a subset of cells display increased biofilm-related gene expression. Induction of *eps* operon expression could potentially be responsible for the observed advancement in pellicle timing by surfactin as aggregates of EPS producers have been found to accelerate biofilm formation [53]. The effect of surfactin on gene expression in aggregates during biofilm formation could further be investigated by fluorescence microscopy using promoter fusions reporting biofilm matrix expression.

The importance of cell density in pellicle formation has been documented in earlier literature where other deficiencies could be complemented by higher starting cell density [12]. Therefore, increased growth properties in the presence of surfactin could be a primary factor in advancing pellicle timing. Surfactin has previously been shown to enhance growth yield in stationary cultures through increased oxygen diffusion in the medium [54]. Tween 80 was additionally shown to have similar effect during aerobic culturing, but without the ability to depolarize cell membranes under anaerobic conditions, which was observed for surfactin, making it a potential candidate to assess whether increased oxygen diffusion alone is enough to advance pellicle formation timing. Increased oxygen diffusion could, besides increasing growth yield, also play a role in aerotaxis, which is yet another important factor contributing to the timing of pellicle formation [12]. Thus, future studies should test supplementation of Tween 80 in the medium and assay also aerotaxis deficient strains.

In summary, our study highlights that pellicle formation of natural *B. subtilis* isolates might differ and delay in pellicle biofilm development could primarily be caused by lack of surfactin production.

## CRediT authorship contribution statement

**Rune Overlund Stannius:** Writing – original draft, Visualization, Validation, Methodology, Investigation, Data curation, Conceptualization. **Sarah Fusco:** Methodology, Investigation. **Michael S. Cowled:** Writing – review & editing, Methodology. **Ákos T. Kovács:** Writing – original draft, Supervision, Resources, Funding acquisition, Conceptualization.

## Declaration of competing interest

The authors declare that they have no known competing financial interests that could have appeared to influence the work reported in this paper. Given his role as Co-Editor in Chief, Ákos T. Kovács was not involved in the peer review of this article and has no access to information regarding its peer review. Full responsibility for the editorial process for this article was delegated to Tom Coenye.

## Declaration of competing interest

The authors declare that they have no known competing financial interests or personal relationships that could have appeared to influence the work reported in this paper.

## Data Availability

Data will be made available on request.
