# Supplementary material for: Surfactin accelerates Bacillus subtilis pellicle biofilm development
Source: Biofilm. 2024 Dec 30;9:100249. doi: 10.1016/j.bioflm.2024.100249 (PMC11754971; doi:10.1016/j.bioflm.2024.100249)
Supplement: Multimedia component 1 [file mmc1.pdf]

Supplementary information

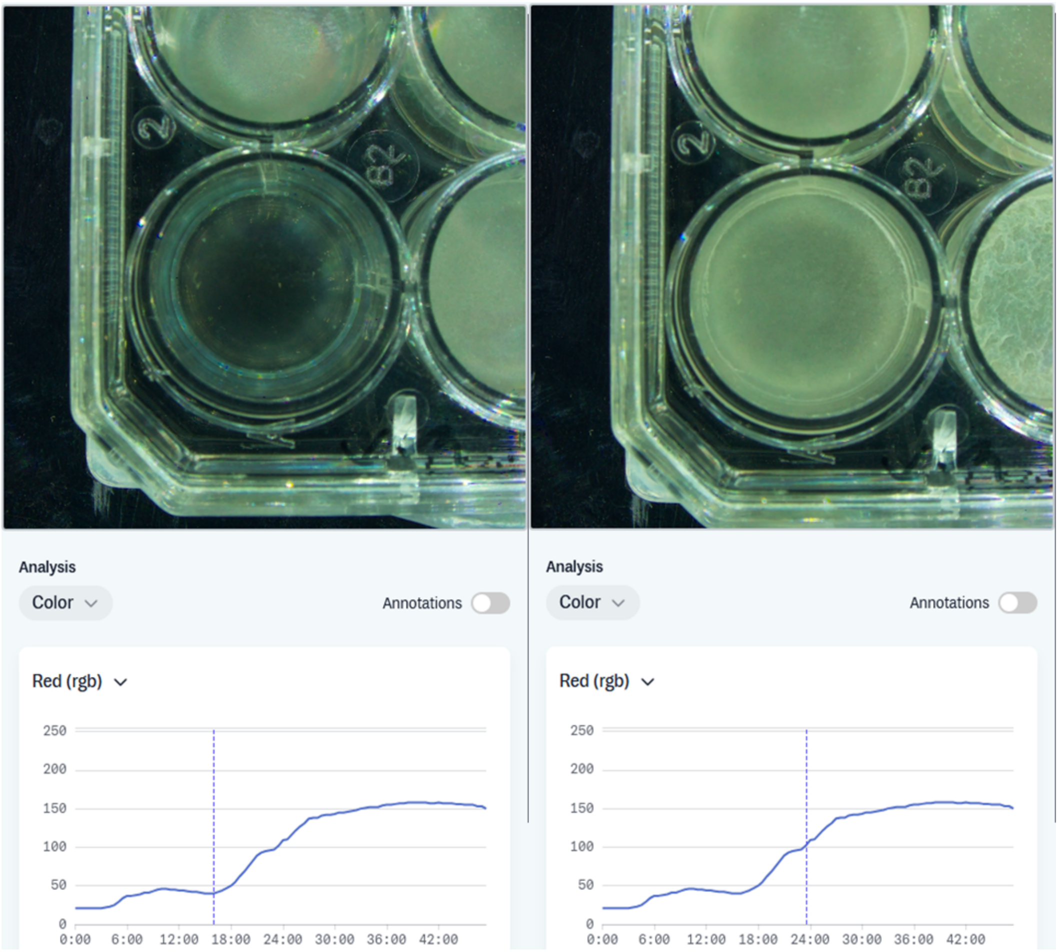

Fig. S1. Screen capture showing pellicle culture of MB8_B1 with color analysis and red value before and at time of pellicle formation.

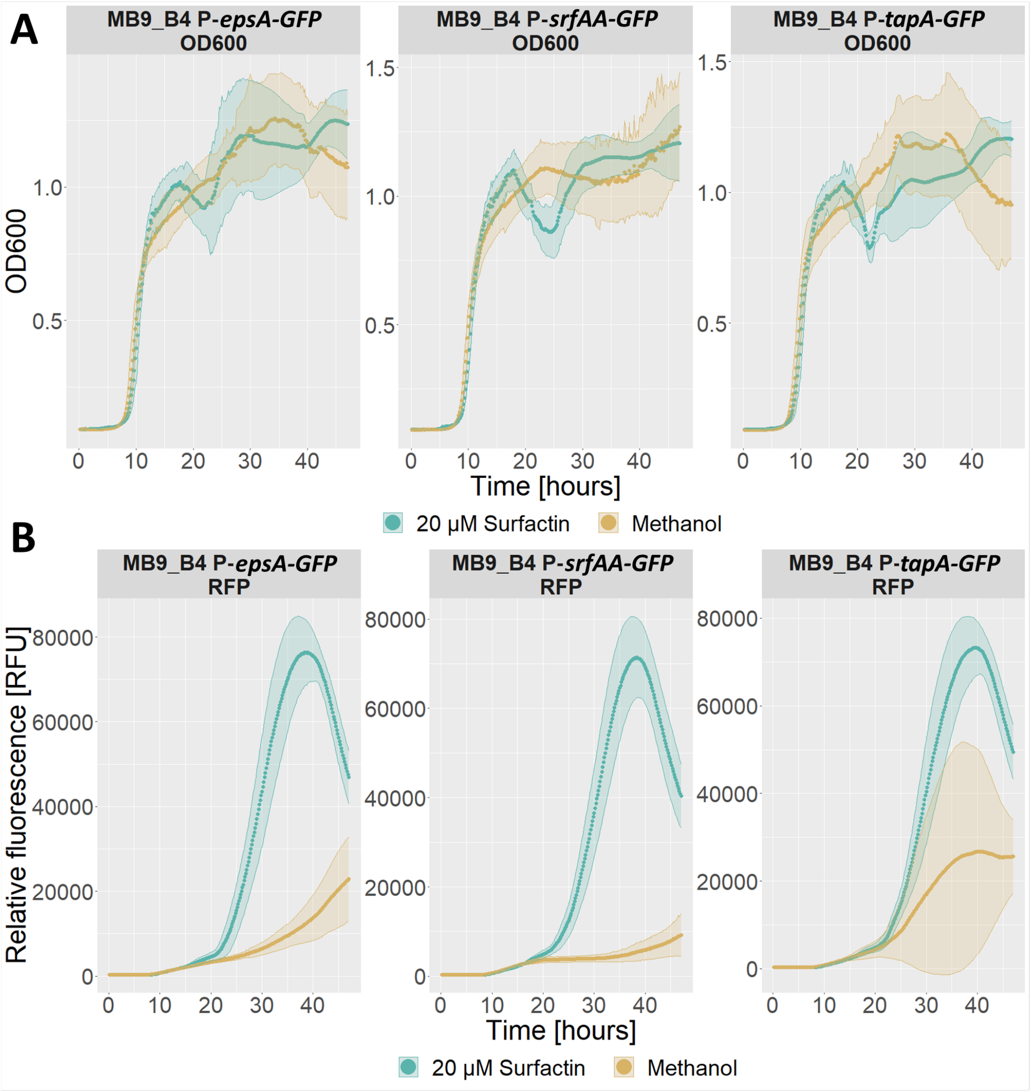

Fig. S2. Effect of addition of surfactin to a final concentration of 20 µM (teal) or pure methanol as a control (beige) on growth and expression of fluorescence under constitutive and promoter-fusion coupled control in MB9_B4 P_{epsA}-*gfp* (left), MB9_B4 P_{srfAA}-*gfp* (middle), and MB9_B4 P_{tapA}-*gfp* (right) over the course of 48 hours in LBgm at 30 °C. A) Optical density at 600 nm. B) Red fluorescence from RFP under control of a constitutive promoter. Data averaged from 3 biological replicates of each strain with ribbons showing standard deviation.
